# Supplementary material for: Sonocatalytic Activity of Porous Carbonaceous Materials for the Selective Oxidation of 4-Hydroxy-3,5-dimethoxybenzyl Alcohol
Source: Molecules. 2024 Mar 23;29(7):1436. doi: 10.3390/molecules29071436 (PMC11013072; doi:10.3390/molecules29071436)
Supplement: Supplementary file 1 [file molecules-29-01436-s001.zip › molecules-2895573-supplementary.pdf]

Supplementary Information

Instrumental and analysis

The N_2 physisorption isotherms were obtained at 77 K using a Micrometrics ASAP 2020 automated system. The samples were dried under a vacuum at 150°C for 6 h before the measurement. The pore size distribution of the materials was calculated from the adsorption branch of the isotherm by Barrett–Joyner–Halenda (BJH) analysis.

XPS analyses were performed in a Microlab 350 (Thermo Electron) equipped with a FEG-tip (Field-Emission Electron Gun) and a twin anode source (AlKa and MgKa). XPS experiments were executed by AlKa (hv = 1486.6 eV) anode X-ray source operated at 15 kV and emission current intensity of 20 mA. The survey and high-resolution spectra from the total surface area of 0.2 cm^2 were recorded using 100 eV and 40 eV pass energy, respectively. Smart background subtraction was applied to obtain XPS signal intensity. The peaks were fitted using an asymmetric Gaussian/Lorentzian mixed function at a constant ratio G/L equal to 0.3 (±0.05). An avantage-based data system software (version 5.9911) was used for processing the analysis. The quantitative chemical composition was estimated using Scofield sensitivity factors.

The FTIR spectra were collected using a Nicolet iS50 (Thermo Scientific) infrared spectrometer equipped with a diamond crystal as a reflection element. The spectra were measured in the range of 4000–400 cm^{-1}. The spectral resolution was set to 4 cm^{-1}. Raman spectra were measured in a 100-3500 cm^{-1} range using a Renishaw InVia Raman spectrometer equipped with a confocal DM 2500 Leica optical microscope, a thermoelectrically cooled CCD as the detector, and Ar laser operating at 514.5 nm. The laser beam was focused on the sample surface using a 20x/0.4 microscope magnification lens. Three 25-second accumulations were taken to acquire all the spectra.

SEM images were obtained from the FEI Nova NanoSEM 230 microscope. The microstructure of the samples was investigated with a Philips CM-20 SuperTwin TEM microscope, operating at 160 kV.

The crystalline structure of the synthesized materials was determined by an X'Pert PRO PANalytical powder diffractometer, Cu Kα radiation. X'Pert HighScore Plus program was used to display and analyze the diffraction patterns, including the full profile fitting procedure. Data were collected in the range of $2\theta=10^{o}-100^{o}$. Thermogravimetric analysis (TGA) was performed in a Derivatograph TG-DTA, MOM Budapest. The samples (50 mg) were heated from room temperature to 800°C with a rate of 5°C min^{-1} under air and then were kept at 800°C for 1h.

For sonocatalytic tests, we use SinapTec generator by applying the frequencies by NexTgen software in different frequencies 22, 100, 500, and 800 kHz 50% of amplitude for each frequency.

The HPLC system that we use is from Waters Company and the system specifications for the Waters Alliance HPLC, Arc HPLC and the ACQUITY UPLC series are as follows :

- Alliance e2695 Separations Module <650 µL (independent of backpressure @ 1.0 mL/min.)
- Acquity UPLC (BSM+FL) : <120 µL (Including a 50 µL mixe)
- Acquity H-Class (QSM+FTN) : <400 uL (Including a 100 µL mixer)
- Acquity I-Class (BSM+FTN) : <100 uL (Including a 50 µL mixer)
- Acquity I-Class (BXM+FL) : <95 µL (Including a 50 µL mixer)

- ACQUITY Arc (QSM-R+FTN-R) : Path 1 ≦ 1450 µL, Path 2 ≦ 1150 µL (Gradient delay volume:Path 1 ≦ 1050 µL, Path 2 ≦ 750 µL), Arc HPLC (QSM-R+FTN-R) : ≦ 1350 µL (Gradient delay volume : ≦ 1000 µL)

EXPERIMENTAL

Catalyst's Characterization

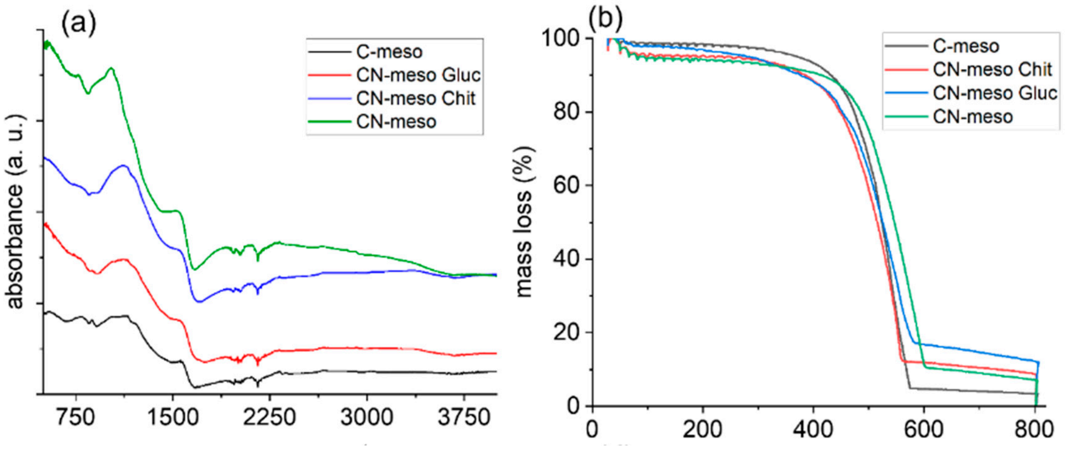

Figure S1 a) The Fourier transform infrared (FTIR), b) TGA analyses

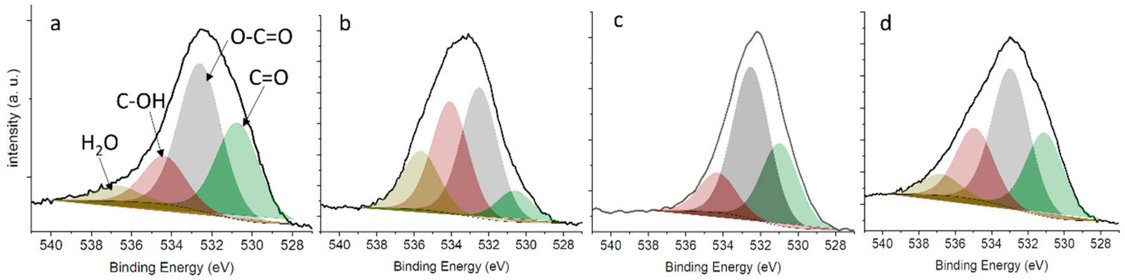

Figure S2. O1s spectra (a) CN-meso Chit (b) CN-meso;(c) CN-meso Gluc; (d) C-meso

Different types of Nitrogen

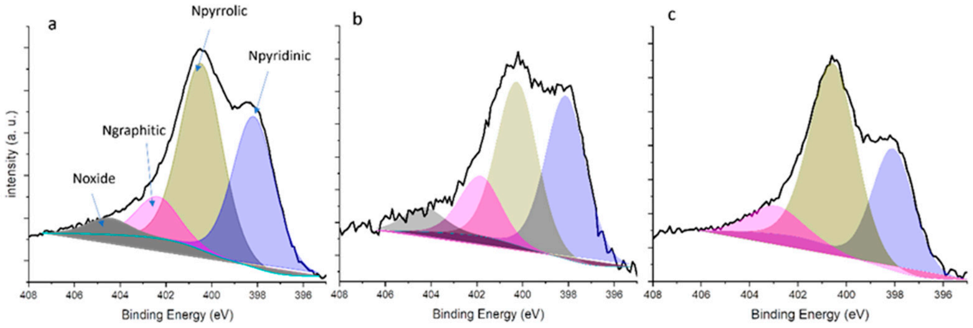

Figure 4 (from min text). N1s (a) CN-meso Chit; (b) CN-meso and (c) CN-meso gluc

Nitrogen, adjacent to carbon in the periodic table, shares a similar atomic size but possesses one additional valence electron. Nitrogen doping can introduce active centers into graphene, suitable for various applications such as electrochemical devices (energy storage and sensors) and spintronics. However, nitrogen can adopt different bonding configurations within the graphene lattice, including pyridinic, pyrrolic, and graphitic forms, as well as adsorbed forms like chemisorbed N and NH2 groups. These various forms exhibit distinct properties, with graphitic nitrogen centers preserving graphene lattice symmetry and electron conjugation. Pyrrolic and pyridinic nitrogen centers possess unique characteristics, with pyridinic nitrogen centers featuring localized lone pairs in non-bonding orbitals [1].

Sonocatalytic test of CN-meso chit, CN-meso gluc, and CN-meso

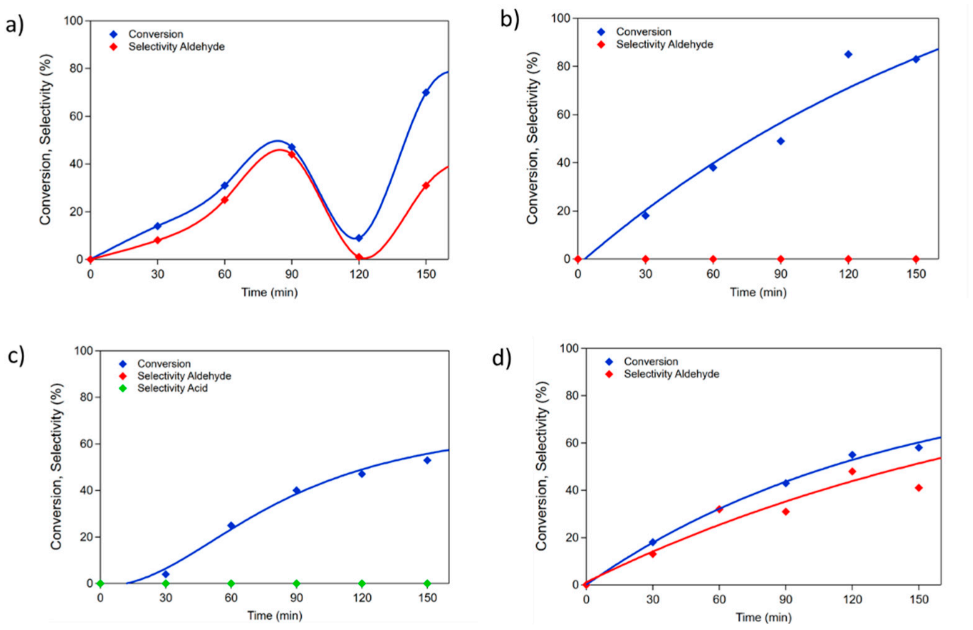

Figure S3 CN-meso Chit sonocatalytic test a) 22kHz, b)100 kHz, c) 500 kHz, and d) 800 kHz

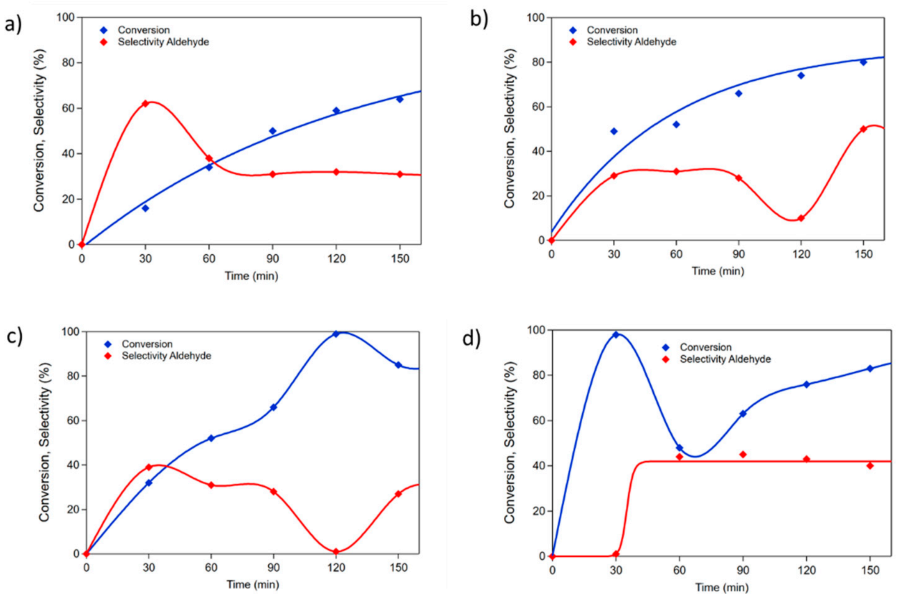

Figure S4 CN-meso Gluc sonocatalytic test a) 22kHz, b)100 kHz, c) 500 kHz, and d) 800 kHz

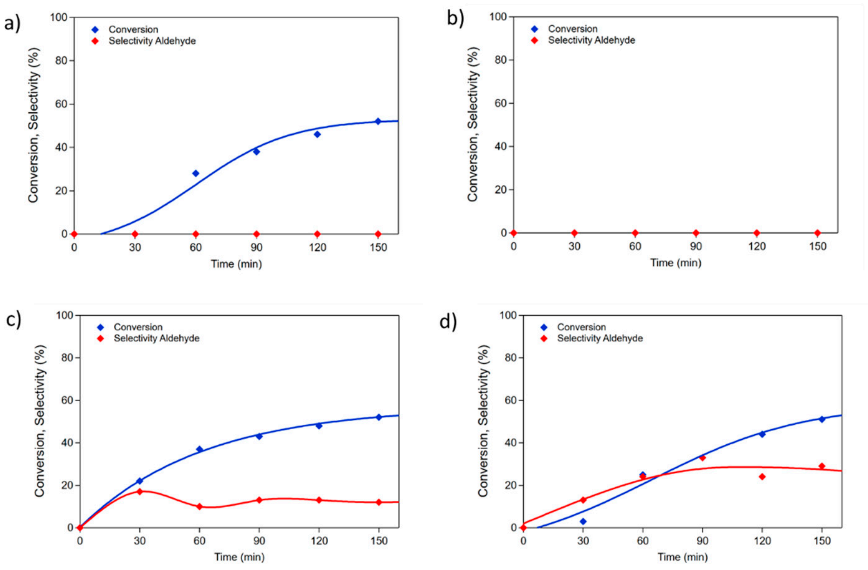

Figure S5 CN-meso sonocatalytic test a) 22kHz, b)100 kHz, c) 500 kHz, and d) 800 kHz

The fluctuations observed in the conversion rate can be explained by considering the influence of the mechanical effects at higher frequencies, combined with the presence of oxidative agents resulting from sonication. At higher frequencies, the predominant effect is the action of the oxidative species on the reaction. Conversely, at lower frequencies, both the mechanical effects and oxidative species contribute to the reaction. This difference in the dominant factors leads to the initial fluctuations in the conversion rate. However, over time, the system reaches a more stable state, indicating that the effects of sonication have been established more consistently.

References Lazar, P., Mach, R., & Otyepka, M. (2019). Spectroscopic fingerprints of graphitic, pyrrolic, pyridinic, and chemisorbed nitrogen in N-doped graphene. The Journal of Physical Chemistry C, 123(16), 10695-10702. doi:10.1021/acs.jpcc.9b02163.
